# PCSK9 and Atrial Fibrillation: A New Perspective on an Old Problem

**DOI:** 10.1155/cdr/7328020

**Published:** 2026-10-26

**Authors:** Amirhossein Sahebkar, Wael Almahmeed, Prashant Kesharwani, Tannaz Jamialahmadi

**Affiliations:** ^1^ Biotechnology Research Center Pharmaceutical Technology Institute School of Pharmacy Mashhad University of Medical Sciences Mashhad Iran mums.ac.ir; ^2^ Centre for Research Impact & Outcome Chitkara College of Pharmacy Chitkara University Rajpura Punjab India chitkara.edu.in; ^3^ Applied Biomedical Research Center Basic Sciences Research Institute Mashhad University of Medical Sciences Mashhad Iran mums.ac.ir; ^4^ Heart and Vascular Institute Cleveland Clinic Abu Dhabi Abu Dhabi UAE clevelandclinicabudhabi.ae; ^5^ Next-Generation Translational Nanomedicine Laboratory Department of Pharmaceutical Sciences Dr. Harisingh Gour Vishwavidyalaya (A Central University) Sagar Madhya Pradesh India; ^6^ University Institute of Pharma Sciences Chandigarh University Mohali Punjab India chandigarhuniversity.ac.in; ^7^ Pharmaceutical Research Center Pharmaceutical Technology Institute School of Pharmacy Mashhad University of Medical Sciences Mashhad Iran mums.ac.ir

**Keywords:** arrhythmia, atrial fibrillation, cholesterol, lipoprotein, oxidative stress, PCSK9, PCSK9 inhibitors, platelet activation, vascular calcification

## Abstract

Proprotein convertase subtilisin/kexin type 9 (PCSK9) has emerged as a novel contributor to atrial fibrillation (AF) pathophysiology. Recent studies suggest that elevated PCSK9 levels may promote AF through multiple mechanisms, including platelet activation, oxidative stress, vascular calcification, and structural atrial remodeling. PCSK9 inhibitors such as alirocumab, evolocumab, and newer agents like inclisiran have shown potential in reducing AF incidence and cardiovascular events, particularly in high‐risk populations. However, evidence from randomized controlled trials with AF as a primary clinical endpoint is limited. Further mechanistic and interventional research is warranted to establish optimized therapeutic strategies targeting PCSK9 in AF prevention and management.

## 1. Introduction

The most prevalent heart arrhythmia, atrial fibrillation (AF), affects millions of people globally. It is linked to considerable morbidity and mortality, especially because of its contribution to the heightened risk of stroke, heart failure, and other cardiovascular events (CVEs) [1, 2]. AF is less prevalent in women than in men; however, women with AF have a higher risk of heart failure, stroke, and mortality [3, 4]. AF represents an increasing global public health burden, with epidemiological studies demonstrating substantial increases in both incidence and prevalence over recent decades and projections suggesting a further rise in absolute disease burden [5]. This calls for the discovery and testing of new therapeutic approaches to reduce disease burden [6–8]. Although AF is generally less prevalent in women than in men, sex‐related differences in clinical outcomes are complex and may be influenced by age, comorbidity burden, treatment patterns, and differences in stroke risk rather than sex alone [9]. Women with AF may experience greater symptom burden and, in several cohorts, a higher risk of thromboembolic events, although contemporary studies have not consistently demonstrated higher mortality or uniformly worse outcomes after adjustment for age and comorbidities [10].

Notwithstanding advancements in treatment, the management of AF remains arduous, particularly in patients with comorbidities such as dyslipidemia and atherosclerosis. In recent years, proprotein convertase subtilisin/kexin type 9 (PCSK9) has been identified as a significant player in cardiovascular diseases (CVDs), and its involvement in AF has attracted growing interest. This review delves into the potential role of PCSK9 in AF and the possible therapeutic implications of PCSK9 inhibition, including alirocumab and evolocumab, in the management of this condition.

## 2. PCSK9 and Its Role in CVDs

PCSK9 is a protein that is essential in lipid metabolism by modulating the degradation of hepatic low‐density lipoprotein receptors (LDLRs). Moreover, several newer physiological roles have also been reported for PCSK9 beyond regulation of cholesterol metabolism [11–13]. Increased PCSK9 levels result in heightened LDL cholesterol (LDL‐C) concentrations, a recognized risk factor for atherosclerotic cardiovascular disease (ASCVD) [14]. PCSK9 inhibitors, including alirocumab and evolocumab, are monoclonal antibodies (mAbs) that attach to PCSK9, inhibiting its interaction with LDLRs and thereby reducing LDL‐C concentrations. These inhibitors are among the newer classes of nonstatin cholesterol‐lowering agents [15, 16] that have demonstrated a substantial reduction in the risk of major adverse cardiovascular events (MACEs) in individuals with dyslipidemia and pre‐existing ASCVD [17]. Beyond its canonical role in LDLR degradation and LDL‐C regulation, PCSK9 has been implicated in inflammatory and vascular pathways involving TLR4/NF‐*κ*B signaling, LOX‐1, macrophage activation, and endothelial dysfunction [18]. Experimental and translational studies suggest that these LDL‐C‐independent effects may contribute to atherosclerosis, thrombosis, and vascular inflammation, although the magnitude and clinical relevance of these pleiotropic actions in humans remain incompletely established [19]. Recent evidence further supports the concept that PCSK9 may exert cardiovascular effects through mechanisms extending beyond LDLR regulation, including inflammatory, prothrombotic, and calcific pathways [20].

## 3. PCSK9 and AF: Emerging Evidence

Recent investigations suggest that PCSK9 may influence AF through both lipid‐dependent and potentially lipid‐independent pathways. Proposed mechanisms include inflammation, oxidative stress, platelet activation, and atrial remodeling; however, the relative contribution and causal relevance of these pathways to AF remain uncertain.

## 4. PCSK9 and AF Risk in the Clinical Setting

A meta‐analysis of 26 randomized controlled trials (RCTs) with 95,635 participants examined the impact of PCSK9 mAbs on the occurrence of AF in individuals at elevated cardiovascular risk. The meta‐analysis found that PCSK9 mAbs were associated with a 16% relative reduction in AF events compared with placebo (RR: 0.84; 95% CI: 0.72–0.98). Although the effect size was modest, the confidence interval excluded the null value; the clinical significance of this finding remains uncertain because AF was generally not a primary endpoint in the included trials. No significant difference was observed between PCSK9 mAbs and ezetimibe (RR: 0.90; 95% CI: 0.29–2.76), with the wide confidence interval including both a potentially substantial reduction and increase in AF risk, indicating considerable statistical uncertainty. Moreover, interpretation of these findings requires caution because the ascertainment and adjudication of AF events may have differed across studies. These findings, whereas hypothesis‐generating, indicate that PCSK9 inhibition may confer a possible association between PCSK9 inhibition and a lower incidence of AF, possibly via mechanisms independent of its putative lipid‐lowering effects [21].

The ODYSSEY OUTCOMES study, which evaluated alirocumab against a placebo in 18,924 patients with recent acute coronary syndrome (ACS) and dyslipidemia, found that alirocumab was not associated with a statistically significant reduction in new‐onset AF (HR: 0.91; 95% CI: 0.77–1.09), corresponding to an estimated 9% relative reduction, but with a confidence interval that included the possibility of no effect or a modest increase in risk. Nonetheless, the research indicated that alirocumab diminished the risk of MACE, irrespective of previous AF history. This indicates that PCSK9 inhibitors could possibly confer cardiovascular advantages in patients with AF by diminishing the risk of MACE [22]. However, treatment with alirocumab or placebo did not influence the cumulative incidence of AF. Although alirocumab did not modify risk of incident AF after ACS, it did reduce the risk of MACE, regardless of prior AF history [22].

There is also a need for additional large‐scale studies looking at the therapeutic impact of PCSK9 inhibitors on AF risk. The EVOCABG study is a Phase III trial that is exploring the cardioprotective effects of preoperative evolocumab administration in patients undergoing elective coronary artery bypass surgery (CABG). Although the primary outcome of this trial is ischemia–reperfusion‐related myocardial injury, AF is prespecified as a secondary outcome [23].

A Mendelian randomization study further provided supportive evidence for a potential association between genetically predicted suppression of PCSK9 and the risk of AF. Genetically predicted suppression of PCSK9 was associated with an estimated 8% lower odds of AF (OR: 0.92; 95% CI: 0.85–0.99). Although the confidence interval narrowly excluded the null value, the modest effect size and the assumptions underlying Mendelian randomization warrant cautious interpretation. Conversely, the inhibition of HMG‐CoA reductase (the pharmacological target of statins) was not found to be linked to the risk of AF. The data indicate that PCSK9 inhibition may confer a specific protective effect against AF, separate from that of statins and mere LDL‐C reduction [24].

Importantly, the available clinical evidence does not consistently support an antiarrhythmic effect of lipid‐lowering therapy. In the ODYSSEY OUTCOMES trial, alirocumab did not significantly alter the incidence of new‐onset AF despite reducing major CVEs, indicating that cardiovascular benefit should not be interpreted as evidence of a direct antiarrhythmic effect [22]. Similarly, evidence regarding statins and AF has been inconsistent. Meta‐analysis of randomized trials has suggested a protective association, whereas larger and longer‐term randomized evidence has not demonstrated a significant reduction in AF [25, 26].

The available clinical evidence linking PCSK9 inhibition with AF should be interpreted according to study design and endpoint hierarchy. In most randomized trials of PCSK9 inhibitors, AF was not a prespecified primary endpoint; therefore, observed differences in AF incidence should generally be considered secondary or exploratory findings rather than definitive evidence of an antiarrhythmic effect. Observational associations between circulating PCSK9 concentrations and AF‐related outcomes may provide biological and prognostic signals but cannot establish causality because PCSK9 levels may reflect systemic inflammation, metabolic dysfunction, atherosclerotic burden, or other cardiovascular comorbidities. Similarly, Mendelian randomization provides supportive evidence regarding potential causality but remains dependent on the validity of genetic instruments and assumptions regarding pleiotropy and pathway specificity.

Consequently, the current clinical evidence should be regarded as hypothesis‐generating, and dedicated randomized studies with AF incidence, AF burden, progression, or recurrence as prespecified endpoints are required to establish a direct antiarrhythmic effect.

Therefore, the apparent association between PCSK9 inhibition and AF should currently be regarded as hypothesis‐generating rather than evidence of an established antiarrhythmic treatment effect (Table 1).

**Table 1:** Clinical studies evaluating PCSK9 inhibition or PCSK9‐related biomarkers and atrial fibrillation outcomes.

Study	Population	PCSK9 intervention/exposure	Effect estimate	Key finding	Ref.
Meta‐analysis of RCTs	Participants at elevated cardiovascular risk	PCSK9 monoclonal antibodies	RR 0.84; 95% CI 0.72–0.98	PCSK9 monoclonal antibody therapy was associated with reduction in AF.	[21]
RCT	Patients with recent ACS and dyslipidemia	Alirocumab vs. placebo	HR 0.91; 95% CI 0.77–1.09	Alirocumab was not significantly associated with AF incidence.	[22]
Mendelian randomization	Genetically characterized population	Genetically predicted PCSK9 suppression	OR 0.92; 95% CI 0.85–0.99	Genetically predicted PCSK9 inhibition was associated with a modest reduction in AF risk.	[24]
Observational study	Patients with AF	Circulating PCSK9	HR 1.64; 95% CI 1.12–2.41	Higher circulating PCSK9 levels were associated with a higher risk of cardiovascular events among patients with AF.	[27]
Post hoc observational analysis	Patients with AF	PCSK9 and LPS levels	—	The study identified an association between PCSK9 and endotoxemia (LPS) in patients with AF, suggesting a potential link between PCSK9 and inflammatory pathways relevant to AF.	[28]
Observational study	Patients with paroxysmal AF	Circulating PCSK9	—	Circulating PCSK9 was investigated as a determinant of AF progression, suggesting that higher PCSK9 may be associated with a more advanced AF phenotype.	[29]
Observational study	Patients with AF	Circulating PCSK9	OR 1.65; 95% CI 1.05–2.60; HR 2.20; 95% CI 1.44–3.37	Higher PCSK9 levels were associated with an abnormally high ABI and increased vascular risk, indicating a relationship between PCSK9 and an adverse vascular phenotype in AF.	[30]

Abbreviations: ABI, ankle–brachial index; ACS, acute coronary syndrome; AF, atrial fibrillation; CABG, coronary artery bypass grafting; CI, confidence interval; CVEs, cardiovascular events; HR, hazard ratio; LPS, lipopolysaccharide; OR, odds ratio; PCSK9, proprotein convertase subtilisin/kexin Type 9; RCT, randomized controlled trial; RR, risk ratio.

## 5. PCSK9 and Platelet Activation

A proposed mechanism connecting PCSK9 to AF is its involvement in platelet activation. A study of 88 patients with AF revealed that individuals with elevated PCSK9 levels (> 1.2 ng/mL) had markedly heightened levels of markers of platelet activation and oxidative stress compared with those with reduced PCSK9 levels [31]. The study suggested that PCSK9 may directly interact with platelet CD36 and enhance platelet activation, with LDL further amplifying this effect. These findings provide a potential mechanistic link between elevated PCSK9 and the prothrombotic state observed in AF [31].

A pertinent investigation found that higher PCSK9 levels were associated with increased urinary 11‐dehydro‐thromboxane B2 (11‐dh‐TxB2), a marker of platelet activation, and a higher risk of CVEs in patients with AF. Patients in the highest PCSK9 tertile had a higher risk of CVEs than those in the lowest tertile (HR: 1.64; 95% CI: 1.12–2.41; *p* = 0.012) [27]. However, because this evidence is observational, it does not establish whether PCSK9 directly promotes platelet activation or instead reflects underlying inflammation and cardiovascular comorbidity.

Taken together, elevated PCSK9 concentrations have been associated with enhanced platelet activation and CVEs in patients with AF, providing a potential mechanistic link between PCSK9 and the prothrombotic phenotype of AF. However, these associations should be interpreted cautiously because PCSK9 concentrations may be influenced by systemic inflammation, dyslipidemia, renal dysfunction, obesity, diabetes, atherosclerotic disease, and other cardiovascular comorbidities that independently affect platelet reactivity and thrombotic risk. Experimental studies indicate that PCSK9 may influence inflammatory and thrombotic signaling, including tissue factor expression through the TLR4/NF‐*κ*B pathway, but these findings do not establish that circulating PCSK9 directly drives platelet activation in patients with AF [32]. Therefore, PCSK9‐associated platelet activation should currently be considered a plausible mechanistic hypothesis rather than an established causal pathway.

## 6. PCSK9 and Atrial Remodeling

Atrial remodeling, defined by adverse electrical and structural alterations, is a critical element in the onset and establishment of AF. A study utilizing a rat model of rheumatoid arthritis (RA), a disorder linked to a heightened risk of AF, revealed that increased levels of PCSK9 correlated with increased susceptibility to AF and atrial structural remodeling. The research indicated that treatment with evolocumab, a PCSK9 inhibitor, decreased AF susceptibility and improved atrial structural remodeling in rats with RA. These findings suggest that PCSK9 may facilitate AF through the promotion of atrial structural remodeling and that PCSK9 inhibition warrants further investigation as a potential strategy for reducing AF susceptibility in high‐risk groups [33]. However, additional research is required to ascertain if these findings are applicable to human patients, especially those with comorbidities such as RA or other inflammatory disorders. Experimental models provide important mechanistic evidence that inflammation‐associated PCSK9 elevation may influence atrial structural remodeling and AF susceptibility; however, extrapolation to human disease should be approached cautiously. Atrial fibrosis and remodeling in human AF are heterogeneous processes influenced by age, hypertension, metabolic disease, ischemia, inflammation, and other comorbidities, which may not be fully reproduced by a single inflammatory animal model [34].

## 7. PCSK9 and Atrial Electrophysiology: Potential Molecular Mechanisms

Beyond its established role in lipid metabolism, PCSK9 may contribute to AF through interconnected inflammatory and oxidative pathways that may ultimately promote atrial remodeling (Figure 1).

**Figure 1:**
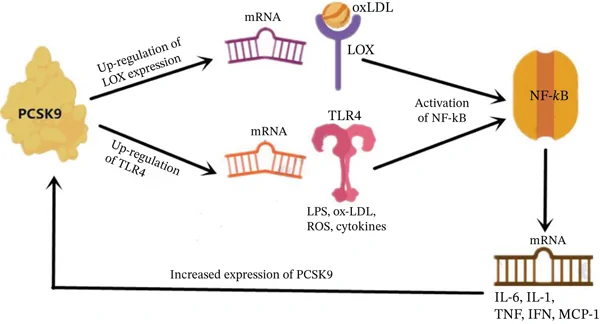
Proposed mechanism underlying the proinflammatory effects of PCSK9. PCSK9 promotes the expression of Toll‐like receptor 4 (TLR4) and lectin‐like oxidized LDL receptor‐1 (LOX‐1), thereby enhancing cellular responses to lipopolysaccharide (LPS) and facilitating the uptake of oxidized LDL (ox‐LDL), respectively. These processes converge on the activation of nuclear factor‐*κ*B (NF‐*κ*B), which subsequently induces the expression of proinflammatory mediators, including interleukin‐1 (IL‐1), interleukin‐6 (IL‐6), tumor necrosis factor‐*α* (TNF‐*α*), interferon‐*γ* (IFN‐*γ*), and monocyte chemoattractant protein‐1 (MCP‐1). Collectively, these pathways may contribute to the amplification of vascular inflammation and atherogenesis. This figure was adapted from reference [19].

Experimental evidence indicates that PCSK9 can activate the TLR4/NF‐*κ*B [35, 36] axis and NLRP3 inflammasome [37], promoting proinflammatory signaling, whereas interaction with CD36 and activation of Nox2 may increase ROS generation [38, 39, 31]. Moreover, PCSK9 upregulated the expression of MyD88, phosphorylated I*κ*B*α*, p65, NLRP3, and IL‐1, whereas inhibition of TLR signaling attenuated these effects. As illustrated in Figure 2, PCSK9 activates the TLR4/MyD88/NF‐*κ*B signaling axis, subsequently promoting NLRP3 inflammasome activation and enhancing profibrotic cellular responses [37]. These pathways may converge on macrophage activation, fibrosis, and cardiomyocyte injury, thereby contributing to an arrhythmogenic atrial substrate.

**Figure 2:**
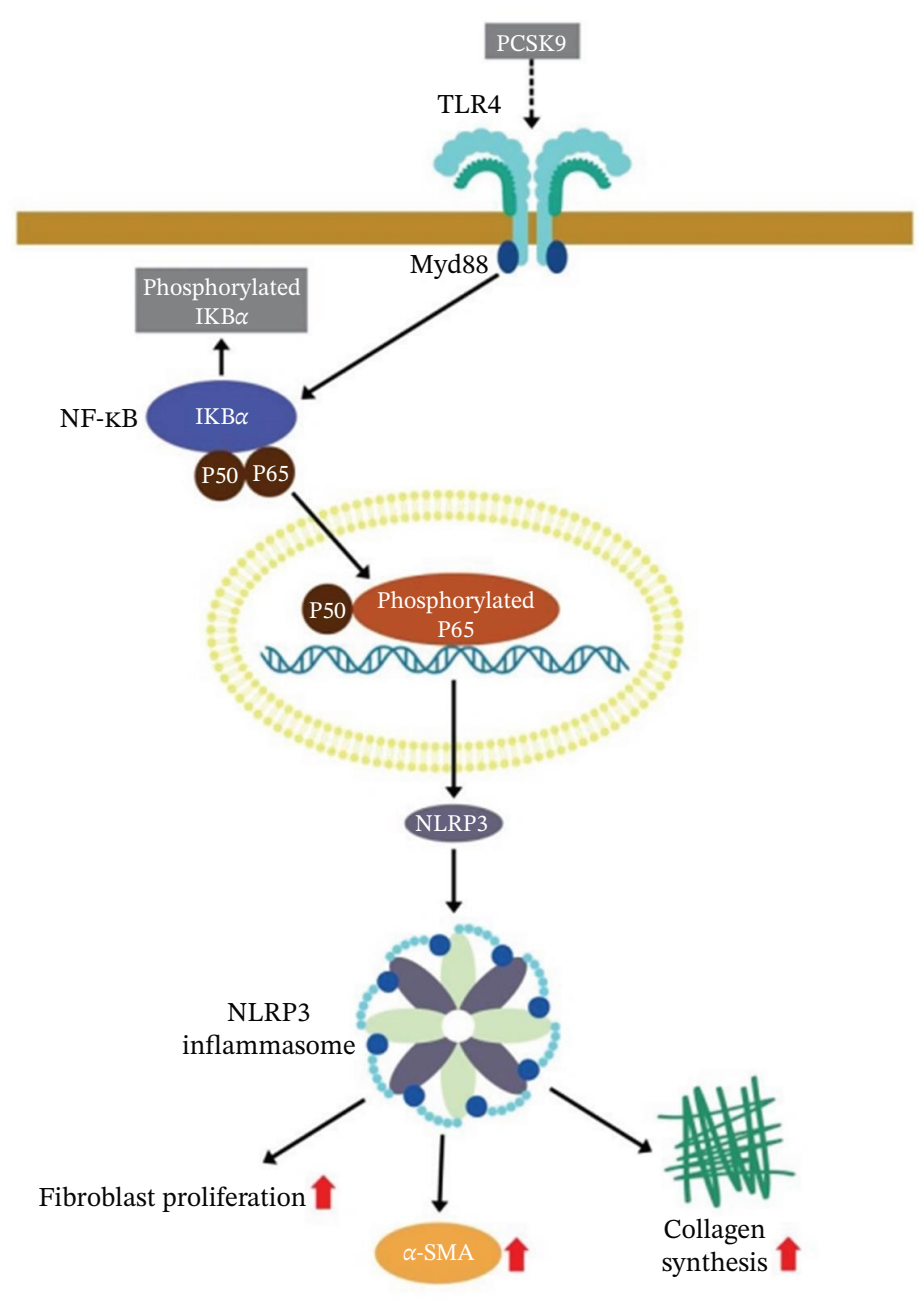
Proposed molecular mechanism underlying the profibrotic effects of proprotein convertase subtilisin/kexin Type 9 (PCSK9) in cardiac fibroblasts. PCSK9 activates the TLR4/MyD88/NF‐*κ*B signaling axis, thereby promoting NLRP3 inflammasome activation and enhancing the profibrotic responses of cardiac fibroblasts. This figure was adapted from reference [37].

Consistent with this hypothesis, an experimental model of inflammation‐associated AF showed that increased PCSK9 promoted M1 macrophage polarization, atrial inflammation, fibrosis, and cardiomyocyte apoptosis, whereas evolocumab attenuated these changes and reduced AF susceptibility (Table 2; Figure 3) [33].

**Table 2:** Proposed mechanistic pathways linking PCSK9 to atrial fibrillation.

Pathway	Proposed mechanism	Potential consequence for AF	Key finding	Key limitation	Ref.
LDLR/LDL‐C pathway	PCSK9 promotes hepatic LDLR degradation and increases circulating LDL‐C	Atherosclerosis, ischemic injury, and cardiovascular remodeling	PCSK9‐mediated lipid elevation provides an established cardiovascular pathway that could indirectly contribute to AF substrate formation.	Direct contribution to AF remains uncertain	[40]
CD36/Nox2 pathway	PCSK9 interacts with CD36 and promotes Nox2‐mediated ROS generation	Oxidative stress and atrial electrical/structural remodeling	PCSK9‐CD36 signaling was linked to enhanced Nox2‐mediated oxidative stress and platelet activation in patients with AF.	Causal relevance to human AF remains unproven	[31]
TLR4/NF‐*κ*B pathway	PCSK9 may activate inflammatory signaling and cytokine production	Atrial inflammation and remodeling	PCSK9 has been implicated in amplification of TLR4/NF‐*κ*B‐dependent inflammatory signaling.	Limited AF‐specific human validation	[39]
NLRP3 inflammasome	PCSK9 may promote inflammasome activation and IL‐1*β*/IL‐18 signaling	Inflammation and fibrosis	PCSK9 was associated with activation of TLR‐related inflammatory signaling and NLRP3 inflammasome pathways.	Direct PCSK9‐dependent effect in human AF remains uncertain	[37]
Platelet activation	PCSK9–CD36 interaction may enhance platelet activation	Thrombogenicity and cardiovascular events	PCSK9 was associated with enhanced platelet activation through CD36/Nox2 signaling.	Confounding by systemic inflammation and cardiovascular disease	[31]
Macrophage activation/fibrosis	PCSK9 may promote M1 polarization and profibrotic signaling	Atrial fibrosis and structural remodeling	In an experimental rheumatoid arthritis model, PCSK9 inhibition with evolocumab attenuated atrial remodeling and reduced AF susceptibility.	Species and model limitations	[33]
Endotoxemia/LPS	PCSK9 is associated with circulating LPS levels	Potential amplification of inflammatory/oxidative pathways	PCSK9 was associated with endotoxemia‐related markers.	Causal PCSK9–LPS–AF pathway has not been established	[28]
Endothelial dysfunction/vascular disease	PCSK9 may contribute to endothelial dysfunction, arterial stiffness, and vascular calcification	Indirect contribution to cardiovascular and atrial substrate	Long‐term PCSK9 inhibition has been associated with improvements in endothelial and vascular function.	Direct link to AF initiation remains uncertain	[41]

Abbreviations: AF, atrial fibrillation; CD36, cluster of differentiation 36; LDL‐C, low‐density lipoprotein cholesterol; LDLR, low‐density lipoprotein receptor; LPS, lipopolysaccharide; NF‐*κ*B, nuclear factor kappa B; NLRP3, NOD‐like receptor family pyrin domain‐containing 3; Nox2, NADPH oxidase 2; PCSK9, proprotein convertase subtilisin/kexin Type 9; ROS, reactive oxygen species; TLR4, Toll‐like receptor 4.

**Figure 3:**
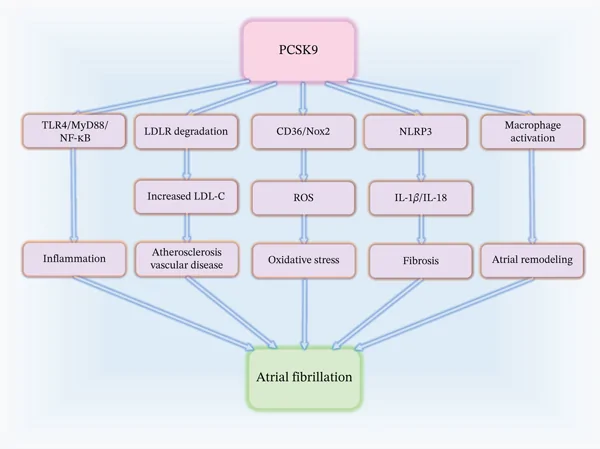
Proposed mechanistic pathways linking PCSK9 to atrial fibrillation (AF). Schematic representation of the potential mechanisms through which proprotein convertase subtilisin/kexin Type 9 (PCSK9) may contribute to AF. PCSK9‐mediated LDL receptor (LDLR) degradation promotes increased circulating LDL‐C and atherosclerotic vascular disease, whereas activation of TLR4/MyD88/NF‐*κ*B signaling contributes to inflammation. In parallel, PCSK9‐associated CD36/NOX2 activation enhances reactive oxygen species (ROS) generation and oxidative stress. Activation of the NLRP3 inflammasome promotes IL‐1*β*/IL‐18 signaling and fibrosis, whereas macrophage activation may facilitate atrial structural remodeling. Collectively, these interconnected inflammatory, lipid‐related, oxidative, fibrotic, and remodeling pathways may promote the development and progression of AF. CD36, cluster of differentiation 36; IL‐1*β*, interleukin‐1 beta; IL‐18, interleukin‐18; LDL‐C, low‐density lipoprotein cholesterol; LDLR, low‐density lipoprotein receptor; MyD88, myeloid differentiation primary response 88; NF‐*κ*B, nuclear factor kappa B; NLRP3, NOD‐like receptor family pyrin domain‐containing 3; NOX2, NADPH oxidase 2; PCSK9, proprotein convertase subtilisin/kexin Type 9; ROS, reactive oxygen species; TLR4, toll‐like receptor 4.

## 8. PCSK9 and Endotoxemia: A Potential Link to AF

Endotoxemia and gut‐derived lipopolysaccharides (LPS) have emerged as potential components of the inflammatory milieu associated with AF and cardiovascular complications. In patients with AF, circulating LPS has been associated with MACEs and with markers of intestinal barrier dysfunction and structural remodeling [42]. Similarly, circulating LPS has been associated with AF recurrence after catheter ablation, suggesting a potential relationship between endotoxemia, systemic inflammation, and atrial remodeling [43]. However, the relationship between PCSK9, LPS, and AF remains mechanistically unresolved, and experimental studies have produced context‐dependent findings regarding the effect of PCSK9 on LPS handling and inflammatory responses [44]. Thus, current evidence supports further investigation of the PCSK9–LPS axis but does not establish a direct causal pathway linking endotoxemia, PCSK9, and AF.

Recent research indicates that endotoxemia, marked by increased concentrations of lipopolysaccharides LPS in the bloodstream, may represent a novel risk factor for CVDs, including AF. A post hoc examination of the ATHERO‐AF cohort revealed a significant correlation between LPS and PCSK9 levels, with patients exhibiting elevated levels of both LPS and PCSK9 facing an augmented risk of CVEs. These findings raise the possibility that PCSK9 and LPS‐related pathways may be interconnected with inflammatory and oxidative processes relevant to AF; however, whether this association contributes causally to AF remains uncertain. Additional research is required to investigate this potential association and its ramifications for the prevention and treatment of AF [28].

## 9. PCSK9 and Vascular Calcification in AF

A separate study examined the correlation between PCSK9 levels and an elevated ankle–brachial index (ABI) in patients with AF. The research indicated that PCSK9 levels correlated with an elevated ABI (≥ 1.4), which is associated with vascular calcification and poorer prognoses. Vascular calcification is an active, cell‐mediated process involving vascular smooth–muscle‐cell phenotypic transition, inflammation, oxidative stress, and extracellular vesicle signaling, and is associated with increased cardiovascular risk [45]. Experimental evidence also suggests that PCSK9 may directly promote vascular smooth‐muscle‐cell calcification and a procalcific phenotype, providing biological plausibility for an association between elevated PCSK9 and vascular calcification [46]. Although vascular calcification is not itself an established direct cause of AF, the associated arterial stiffness, endothelial dysfunction, inflammation, and hemodynamic load may contribute to atrial cardiomyopathy and remodeling, thereby potentially facilitating an arrhythmogenic substrate [47].

Patients with PCSK9 levels > 1150 pg/mL had approximately 65% higher odds of an abnormal ABI (OR: 1.65; 95% CI: 1.05–2.60). Although the confidence interval was above the null value, the magnitude of the association should be interpreted cautiously because the study was observational. PCSK9 levels > 2060 pg/mL were associated with more than twofold higher hazard of all‐cause mortality (HR: 2.20; 95% CI: 1.44–3.37). Although the confidence interval indicates a statistically significant association, this observational finding should not be interpreted as evidence of a causal effect of PCSK9 on mortality. The data suggest that PCSK9 may have a role in vascular calcification among individuals with AF, hence emphasizing its significance in cardiovascular pathology [30].

## 10. PCSK9 and AF Progression

The progression of AF from paroxysmal to more persistent forms is a notable clinical concern. A study investigating the prevalence and drivers of AF progression in paroxysmal atrial fibrillation (PAF) discovered multiple biomarkers linked to AF progression, notably elevated levels of PCSK9. The study demonstrated that PCSK9, along with other markers such as N‐terminal probrain natriuretic peptide and tissue factor pathway inhibitor, was associated with an increased risk of AF exacerbation. This suggests that PCSK9 may play a role in the progression of AF, potentially through mechanisms associated with atrial remodeling, inflammation, and coagulation [29]. In patients with paroxysmal AF, elevated PCSK9 was associated with AF progression, supporting a potential role of PCSK9 in disease progression rather than establishing a uniform effect across all AF phenotypes [29].

## 11. PCSK9 and AF Phenotypes

AF should not be considered a homogeneous clinical entity, and the relationship between PCSK9 and AF may differ according to AF phenotype and underlying substrate. Available clinical evidence has primarily evaluated AF as a composite outcome or studied patients with established nonvalvular AF, limiting phenotype‐specific conclusions. In patients with paroxysmal AF, PCSK9 was independently associated with AF progression, suggesting that PCSK9 may be more relevant to the transition from paroxysmal to persistent or permanent AF than to AF initiation alone [29]. In contrast, direct evidence linking PCSK9 to persistent/permanent AF, postoperative AF, or AF specifically associated with structural heart disease remains limited. Experimental studies suggesting effects of PCSK9 on inflammation, oxidative stress, fibrosis, and atrial remodeling may be particularly relevant to inflammation‐associated and structurally mediated AF, but these findings cannot yet be extrapolated to all clinical phenotypes. Therefore, future studies should evaluate whether PCSK9 is associated differently with AF initiation, progression, recurrence, and postoperative AF.

## 12. Lipid‐Dependent and Lipid‐Independent Mechanisms of PCSK9 in AF

PCSK9 has also been implicated in inflammatory signaling, oxidative stress, and platelet activation through pathways involving TLR4/NF‐*κ*B, NLRP3, CD36, and Nox2. As discussed above, these mechanisms may interact with rather than act independently of lipid metabolism, and their contribution to AF remains incompletely established. In vitro studies using siRNA‐mediated SDC4 silencing in primary macrophages provided evidence that SDC4 interacts with PCSK9 in vivo and contributes to the proinflammatory responses elicited by PCSK9 (Figure 4) [48].

**Figure 4:**
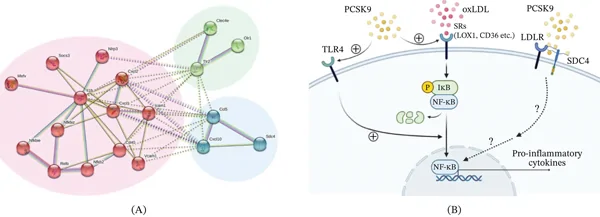
Lipid‐ and LDL receptor‐independent proinflammatory actions of PCSK9 in macrophages. (A) STRING‐based network analysis of the 24 overrepresented transcripts identified in Ldlr−/− mouse macrophages. Nodes are color‐coded according to k‐means clustering (three clusters), and only interconnected nodes are displayed. The panel was reproduced from Katsuki et al. with permission from the publisher. (B) Proposed signaling mechanisms underlying PCSK9‐mediated macrophage activation. Binding of oxidized LDL (oxLDL) to scavenger receptors, including LOX‐1 and CD36, promotes I*κ*B phosphorylation and degradation, thereby facilitating NF‐*κ*B activation. PCSK9 may potentiate this inflammatory pathway by increasing the expression of scavenger receptors and TLR4. In addition, direct interaction between SDC4 and PCSK9 may promote the production of proinflammatory cytokines, potentially through activation of the NF‐*κ*B signaling pathway. This figure was adapted from reference [48].

PCSK9 has also been associated with endothelial dysfunction and vascular inflammation (Figure 5), which may in turn contribute to arterial stiffness. Clinical data suggest that PCSK9 inhibition may improve endothelial and microvascular function beyond the magnitude expected from LDL‐C reduction alone [41]. These potentially lipid‐independent effects may contribute to the arrhythmogenic substrate through interactions among inflammation, oxidative stress, thrombogenicity, endothelial dysfunction, and atrial remodeling. Nevertheless, current evidence does not establish that these pleiotropic effects independently reduce AF risk in humans. Thus, the relationship between PCSK9 and AF is more appropriately viewed as a multifactorial interaction between LDL‐C‐dependent cardiovascular risk reduction and potentially LDL‐C‐independent inflammatory, oxidative, platelet, and endothelial pathways, and dedicated randomized studies are required to determine the relative contribution of each mechanism.

**Figure 5:**
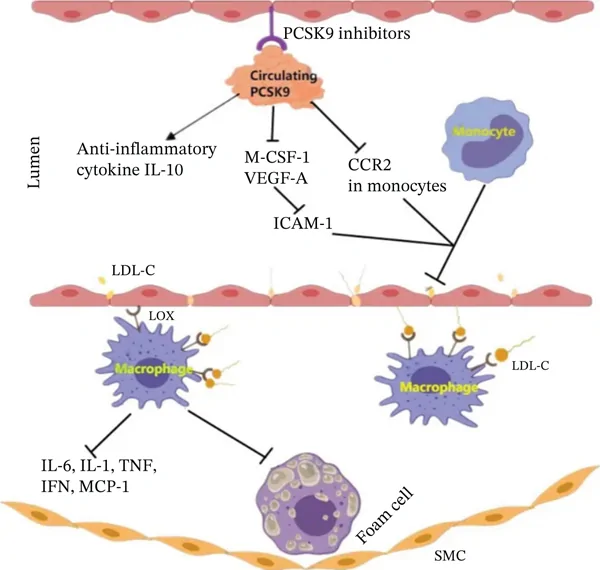
Potential effects of PCSK9 inhibition on vascular inflammation. Inhibition of PCSK9 may attenuate vascular inflammation by reducing the expression of key proinflammatory mediators, including M‐CSF and VEGF‐A, thereby limiting ICAM‐1 expression in endothelial cells and subsequent monocyte recruitment and infiltration into the subendothelial space. PCSK9 inhibition may also suppress CCR2 expression, consequently reducing monocyte chemotaxis and migration. In addition to attenuating proinflammatory signaling, PCSK9‐targeted therapies may exert anti‐inflammatory effects by enhancing the production of anti‐inflammatory cytokines, such as IL‐10. CCR2, C–C chemokine receptor Type 2; ICAM‐1, intercellular adhesion molecule 1; LDL‐C, low‐density lipoprotein cholesterol; LOX‐1, lectin‐like oxidized LDL receptor‐1; MCSF‐1, macrophage colony‐stimulating factor 1; oxLDL, oxidized low‐density lipoprotein; SMCs, smooth muscle cells; VEGF‐A, vascular endothelial growth factor A. The signal “→” indicates “promotes,” “⊣” indicates “inhibits”. This figure was adapted from reference [19].

## 13. Clinical and Translational Implications

The current evidence does not support the use of PCSK9 inhibitors specifically for AF prevention or rhythm control. In patients with AF who have established ASCVD or other indications for intensive LDL‐C lowering, PCSK9 inhibitors remain an evidence‐based strategy for reducing cardiovascular risk; however, whether these agents provide an additional antiarrhythmic benefit remains uncertain. In particular, randomized data have not demonstrated improvements in sinus‐rhythm maintenance, AF burden, cardioversion success, or postablation outcomes. The neutral effect of alirocumab on incident AF in ODYSSEY OUTCOMES further argues against interpreting the cardiovascular benefits of PCSK9 inhibition as evidence of a direct antiarrhythmic effect [22]. Circulating PCSK9 may nevertheless represent a potential biomarker candidate. Higher PCSK9 concentrations have been associated with CVEs and platelet activation in patients with AF, and PCSK9 has also been associated with progression of paroxysmal AF [27, 29]. However, these findings remain observational and do not establish that PCSK9 measurement provides sufficient incremental predictive value to justify routine clinical testing.

Therefore, the most clinically relevant population for PCSK9 inhibition at present comprises patients with AF who already have established indications for intensive lipid lowering, particularly those with ASCVD or very high cardiovascular risk. Whether patients with AF without ASCVD benefit from PCSK9 inhibition specifically with respect to AF incidence or progression remains unknown. Future trials should therefore determine whether PCSK9 inhibition can reduce AF burden or progression in predefined AF phenotypes and whether circulating PCSK9 can be used to identify patients most likely to benefit from treatment.

## 14. Strengths and Limitations of the Current Evidence

Current evidence linking PCSK9 to AF includes randomized trials, Mendelian randomization studies, observational biomarker studies, and experimental models, providing biological plausibility but also important limitations. Evidence from randomized trials is limited because AF was not a primary endpoint in most studies, and differences in event ascertainment, endpoint definitions, and statistical power may affect the findings. Although a meta‐analysis of 26 randomized trials suggested a possible reduction in AF events, the modest effect size and potential heterogeneity limit firm conclusions. Across the available studies, effect estimates should also be interpreted in the context of their confidence intervals, absolute event rates, endpoint definitions, and study design because a statistically significant relative association does not necessarily translate into a clinically meaningful reduction in AF burden or CVEs. Observational studies are also vulnerable to confounding and reverse causation, as elevated PCSK9 may reflect inflammation, metabolic dysfunction, or cardiovascular comorbidities associated with AF. Mendelian randomization studies provide supportive evidence but depend on the validity of genetic instruments and assumptions such as the absence of substantial horizontal pleiotropy. Mechanistic and animal studies suggest potential roles for inflammation, oxidative stress, platelet activation, and atrial remodeling, but their relevance to human AF remains uncertain. Overall, current evidence is hypothesis‐generating, and dedicated prospective studies and randomized trials with AF as a prespecified or primary endpoint are needed to establish whether PCSK9 inhibition can reduce AF incidence or progression. Importantly, the absence of AF‐specific primary endpoints in major PCSK9 inhibitor trials and the neutral findings of ODYSSEY OUTCOMES substantially limit causal inference regarding a direct antiarrhythmic effect of PCSK9 inhibition.

## 15. Plasminogen (PLG) as a Shared Therapeutic Target for AF and Myocardial Infarction (MI)

A Mendelian randomization study found PLG to be a shared protective factor against MI and AF. The research indicated that PLG activators, including urokinase and alteplase, may function as prospective treatment agents for both disorders. The study also revealed PCSK9 as a plasma protein with pleiotropic effects on CVDs, highlighting its potential significance in the pathogenesis of both AF and MI [49].

## 16. Conclusion

The involvement of PCSK9 in AF is increasingly being investigated, with new evidence indicating that PCSK9 may play a role in AF through processes such as platelet activation, oxidative stress, atrial remodeling, and vascular calcification. PCSK9 inhibitors, including alirocumab and evolocumab, have demonstrated cardiovascular benefits in high‐risk populations, while emerging evidence suggests that PCSK9 inhibition may also be associated with a lower risk of AF. However, evidence regarding AF remains limited, as most available data are derived from secondary or post hoc analyses, observational studies, Mendelian randomization analyses, and preclinical models, and dedicated randomized trials with AF as a primary endpoint are lacking. Current evidence suggests that PCSK9 may contribute to AF‐related pathophysiology through interconnected lipid‐dependent and potentially lipid‐independent pathways involving inflammation, oxidative stress, platelet activation, vascular dysfunction, and atrial remodeling. Experimental studies provide biological plausibility, whereas clinical and genetic studies suggest an association between PCSK9‐related pathways and AF risk or progression; however, these findings do not yet establish a direct antiarrhythmic effect of PCSK9 inhibition. The available evidence is therefore best considered hypothesis‐generating, and prospective randomized studies with AF‐specific endpoints are required to determine whether PCSK9 inhibition can meaningfully reduce AF incidence, burden, progression, or recurrence. Further research is necessary to comprehensively clarify the mechanisms underpinning the link between PCSK9 and AF, as well as to ascertain the best application of PCSK9 inhibitors in the prevention and management of AF. In this context, the impact of newer PCSK9 inhibitors like inclisiran as well as investigational peptides and small molecules [50–52] on the pathophysiological features of AF merits further exploration.

In conclusion, PCSK9 represents a plausible and potentially important therapeutic target in AF, and PCSK9 inhibitors may offer a novel approach to mitigate the burden of this common and devastating arrhythmia. Nonetheless, there is a scarcity of evidence from RCTs examining the effect of PCSK9 inhibitors on AF as a primary endpoint. With accumulating evidence of PCSK9′s potential association with AF‐related pathways and disease progression, innovative strategies for the prevention and treatment of AF are anticipated, which can improve patient outcomes worldwide.

## Author Contributions

All authors contributed significantly to this work.

## Funding

This study is based upon research funded by Iran National Science Foundation (INSF) under Project No. 4026662 (grant awarded to A.S.).

## Disclosure

All authors finalized and approved the manuscript for publication.

## Conflicts of Interest

The authors declare no conflicts of interest.

## Data Availability

Data sharing is not applicable to this article as no datasets were generated or analyzed during the current study. All of the relevant data are included within the paper itself.
